# Supplementary material for: Hematopoietic stem-cell gene therapy is associated with restored white matter microvascular function in cerebral adrenoleukodystrophy
Source: Nat Commun. 2023 Apr 5;14:1900. doi: 10.1038/s41467-023-37262-w (PMC10076264; doi:10.1038/s41467-023-37262-w)
Supplement: Supplementary file 1 — Supplementary Information [file 41467_2023_37262_MOESM1_ESM.pdf]

Supplementary Figure 1

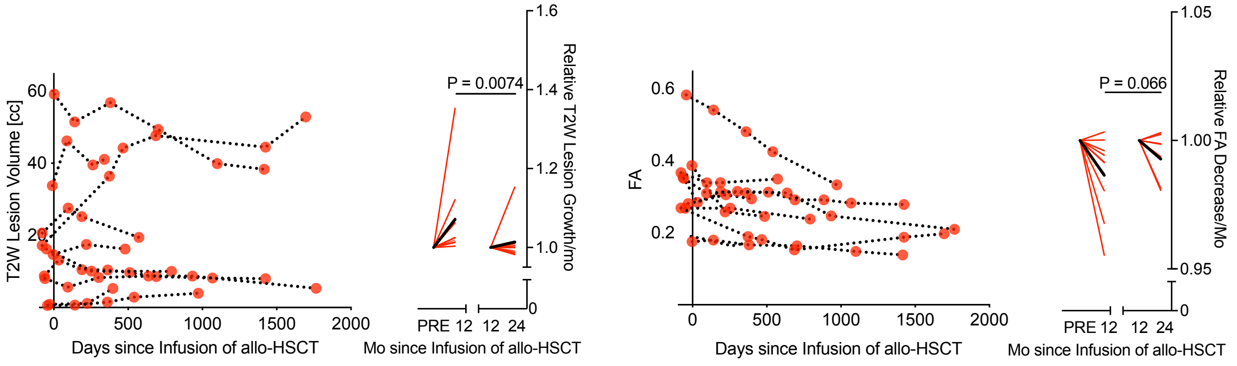

Supplementary Figure 1: Treatment effects after allogeneic hematopoietic stem cell transplantation **(**allo-HSCT). Longitudinal data for T2W lesion volume (left) and mean lesional fractional anisotropy (FA, right) of patients treated with allo-HSCT (n=10). For each left diagram shows the longitudinal course and right diagram shows relative monthly change in lesion volume and FA PRE to within the first- and in the second-year follow up (1-12 Mo, 12-24 Mo) post treatment. The P-values were determined by two-tailed Mann-Whitney test and two-tailed unpaired students t-tests. Source data are provided as a Source Data file.

Supplementary Figure 2

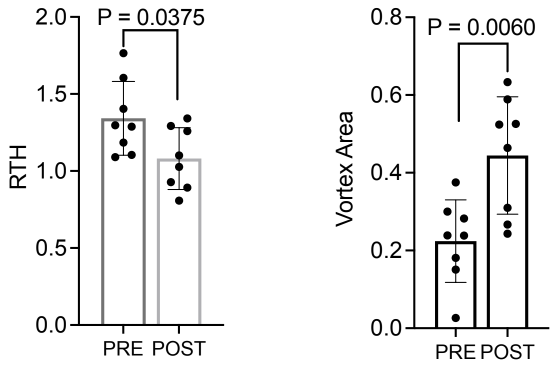

Supplementary Figure 2: Vascular efficiency after allogenic hematopoietic stem cell transplantation (allo-HSCT). Comparison of the mean relative transit time heterogeneity (RTH) and vortex area in normal appearing white matter in patients (n=8) before (PRE) and two years after (POST) treatment allo-HSCT. Data are presented as the mean +- SD. The P-values were determined by paired two-tailed students t-test. Source data are provided as a Source Data file.

Supplementary Figure 3

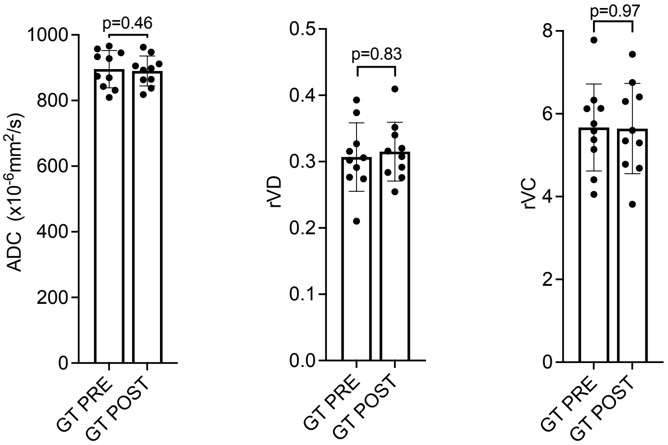

Supplementary Figure 3: Microstructural integrity of normal appearing white matter (NAWM). Mean apparent diffusion coefficient (ADC), relative vessel density (rVD), relative mean vessel caliber (rVC) in NAWM corresponding to perfusion measurements illustrated in figure 3 in CALD patients (n=13) before (PRE) and two years POST gene therapy (GT). Data are expressed as mean+-SD; two-tailed unpaired students t-tests. Source data are provided as a Source Data file.

Supplementary Figure 4

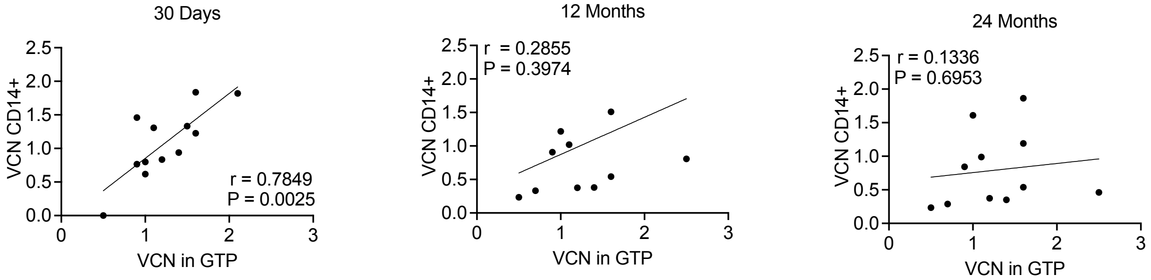

Supplementary Figure 4: Correlations with Vector copy number numbers (VCN) in the gene therapy product. Correlation plots showing relationship between post treatment VCN in CD14+ cells and VCN in the gene therapy product (GTP, n=10-12, line indicates regression; the P-values were determined by two-tailed Pearson's correlation). Source data are provided as a Source Data file.

Supplementary Table 1

Supplementary Table 1: Baseline Characteristics of male ALD Patients with DSC MR Perfusion					
	GT (n=15)	allo-HSCT (n=10)	SA (n=8)	HEM (n=22)	p value*
Age at Treatment, years, median, (range)	7.8 (4.1-8.6)	8.61 (4.8-36.9)	-	-	0.041
Age at first MRI, years, median, (range)	-	-	31.4 (10.4-47.3)	4.2 (0.28-46.8)	
Baseline Loes Score, median (range)	1 (1-2)	5.75 (1 – 9)	3 (1-12)		0.024
Baseline CALD Lesion volume, median cc (interquartile range)	3.84 (1.45-10.72)	15.46 (6.23-23.93)	8.9 (6.8-16.51)	-	0.029
Baseline FA in CALD lesion, mean ± standard deviation	0.36 ± 0.09	0.31 ± 0.11	0.32 ± 0.06	-	0.210
Follow up visits, number (range)	3 (1-7)	3 (1-7)	2 (0-9)	1.5 (0-12)	0.531
Follow up time, days, median (range)	735 (47-1426)	882 (88-1765)	950 (0-3894)	685 (0-2991)	0.253
Number of Scans with Dual Echo Protocol, number (%)	46 (58)	32 (55)	15 (83)	43 (57)	0.886

Supplementary table 1: Baseline Characteristics of male ALD Patients with DSC MR Perfusion. Abbreviations: CALD= inflammatory cerebral demyelination in x-linked adrenoleukodystrophy, FA= fractional anisotropy, GT = gene therapy; allo-HSCT =Allogeneic hematopoietic stem cell transplantation; SA = Self arrested patients with brain lesions consistent of CALD without signs of lesional activity and without specific treatment; HEM = X-linked adrenoleukodystrophy patients without CALD. * represents GT vs. allo-HSCT. P-values were determined by two-tailed Mann-Whitney tests, two-tailed unpaired students t-tests and two-tailed Fisher's test. Source data are provided as a Source Data file.
