# Supplementary material for: Hematopoietic stem-cell gene therapy is associated with restored white matter microvascular function in cerebral adrenoleukodystrophy
Source: Nat Commun. 2023 Apr 5;14:1900. doi: 10.1038/s41467-023-37262-w (PMC10076264; doi:10.1038/s41467-023-37262-w)
Supplement: Supplementary file 3 — Reporting Summary [file 41467_2023_37262_MOESM3_ESM.pdf]

nature portfolio

Corresponding author(s): Dr. Patricia L. Musolino

Last updated by author(s): Jan 10, 2023

Reporting Summary

Nature Portfolio wishes to improve the reproducibility of the work that we publish. This form provides structure for consistency and transparency in reporting. For further information on Nature Portfolio policies, see our Editorial Policies and the Editorial Policy Checklist.

Statistics

For all statistical analyses, confirm that the following items are present in the figure legend, table legend, main text, or Methods section.

n/a	Confirmed	
☐	☒	The exact sample size (*n*) for each experimental group/condition, given as a discrete number and unit of measurement
☐	☒	A statement on whether measurements were taken from distinct samples or whether the same sample was measured repeatedly
☐	☒	The statistical test(s) used AND whether they are one- or two-sided<br>*Only common tests should be described solely by name; describe more complex techniques in the Methods section.*
☐	☒	A description of all covariates tested
☐	☒	A description of any assumptions or corrections, such as tests of normality and adjustment for multiple comparisons
☐	☒	A full description of the statistical parameters including central tendency (e.g. means) or other basic estimates (e.g. regression coefficient) AND variation (e.g. standard deviation) or associated estimates of uncertainty (e.g. confidence intervals)
☐	☒	For null hypothesis testing, the test statistic (e.g. *F*, *t*, *r*) with confidence intervals, effect sizes, degrees of freedom and *P* value noted<br>*Give P values as exact values whenever suitable.*
☒	☐	For Bayesian analysis, information on the choice of priors and Markov chain Monte Carlo settings
☒	☐	For hierarchical and complex designs, identification of the appropriate level for tests and full reporting of outcomes
☐	☒	Estimates of effect sizes (e.g. Cohen's *d*, Pearson's *r*), indicating how they were calculated

Our web collection on statistics for biologists contains articles on many of the points above.

Software and code

Policy information about availability of computer code

Data collection	- PENGUIN perfusion software v1.0 (www.cfin.au.dk/software/penguin).<br>- Vessel architectural imaging analysis was performed using custom made software (Emblem, K. E. et al. Vessel architectural imaging identifies cancer patient responders to anti-angiogenic therapy. Nature medicine 19, 1178–1183; 10.1038/nm.3289 (2013) in MATLAB vR2019a (www.matlab.com)<br>- Image co-registered to structural MRI images using an attribute-based image registration algorithm: DRAMMS v1.5.1 (https://github.com/ouyangming/DRAMMS).<br>- Regions of Interest and output was created with 3D-SLICER v4.6.2 (http://www.slicer.org).<br>- Age-matching was performed using the R package MatchIt v4.5.0 in R v3.5.2
Data analysis	Statistical analysis was performed using SPSS 22.0 (https://www.ibm.com/analytics/spss-statistics-software), Graphpad Prism v9.5.0 (https://www.graphpad.com/scientific-software/prism) and R v3.5.2 (https://cran.r-project.org/bin/windows/base).

For manuscripts utilizing custom algorithms or software that are central to the research but not yet described in published literature, software must be made available to editors and reviewers. We strongly encourage code deposition in a community repository (e.g. GitHub). See the Nature Portfolio guidelines for submitting code & software for further information.

Data

Policy information about availability of data

All manuscripts must include a data availability statement. This statement should provide the following information, where applicable:

- Accession codes, unique identifiers, or web links for publicly available datasets
- A description of any restrictions on data availability
- For clinical datasets or third party data, please ensure that the statement adheres to our policy

STARBEAM study related data that supports the findings of this study are available from Bluebird Bio. Restrictions apply to the availability of these data because elements of the data set comprise information proprietary to Bluebird Bio. Any requests for additional data will be considered by all authors and Bluebird Bio. Bluebird requires 30 days from receipt date of the request to consider and respond to data requests. Any requests can be sent to medinfo@bluebirdbio.com. Data not associated with the STARBEAM study and collected at MGH can be shared with interested investigators but are subject to local and national ethics regulations and legal requirements that respect the informed consent forms. The raw imaging data are protected and are not available due to data privacy laws. The data generated in this study are provided in the Source Data file.

Human research participants

Policy information about studies involving human research participants and Sex and Gender in Research.

Reporting on sex and gender	Adrenoleukodystrophy (ALD) is a disease linked to the X chromosome. The cerebral phenotype affects male sex. Only patients with male sex were investigated. Gender is not reported in our study.
Population characteristics	STARBEAM study: Males aged 17 years and younger (range 4.1 – 8.6 years) who have been definitively diagnosed with CALD (by finding elevated levels of VLCFA) who have a MRI Loes score between 0.5 and 9 (inclusive), an NFS ≤ 1, and gadolinium enhancement on MRI. Patients outside of the STARBEAM trial: Males (age range 0.3-47.3 years) diagnosed with ALD who have a MRI Loes score between 0 and 9 (inclusive at baseline) and available DSC MRI Perfusion imaging as part of routine clinical care.
Recruitment	Participants were recruited by peer to peer physician referral (this study included only boys with no related and no unrelated compatible HSCT donor and therefore have not access to standard of care with allo-HSCT for CALD), referral by Leukodystrophy advocacy groups across the world, self-referral to Dr. Musolino, Dr. Duncan and Dr. Eichler clinics or the sponsor. Open recruitment for the study was published in clinical trials.gov and sponsor websites during the duration of the study. Potential biases are access to internet, ability to speak English by referring team or patient (despite availability of translators to all languages) and inability to travel to the study sites for the screening, treatment and follow up visits. All participants were enrolled after screening multi-PI calls that confirm eligibility on a first come first serve basis. Priority was given to participants with more advance lesions when possible if study drug was available.
Ethics oversight	The study received ethical approval by the Institutional Review Board of MGH (MGH protocol 2012-P-000132/1). Informed consent was obtained from patients and/or from legal representatives.

Note that full information on the approval of the study protocol must also be provided in the manuscript.

Field-specific reporting

Please select the one below that is the best fit for your research. If you are not sure, read the appropriate sections before making your selection.

☒ Life sciences ☐ Behavioural & social sciences ☐ Ecological, evolutionary & environmental sciences

For a reference copy of the document with all sections, see nature.com/documents/nr-reporting-summary-flat.pdf

Life sciences study design

All studies must disclose on these points even when the disclosure is negative.

Sample size	By using the means and common standard deviation for ADC, CTH, Kapp, RTH and VA from pilot data and assuming a two-sided alpha-level of 0.05, homogeneous variances for the samples to be compared and a 80.0% power we calculated that small effect sizes can be detected with lesion size and diffusion tensor data (Hedges' g= 0.11) and large effect sizes with perfusion based data (Hedges' g= 1.04-1.25) for the numbers of available patients.
Data exclusions	Incomplete magnetic resonance perfusion datasets or those significantly degraded by motion artifacts were excluded from the analysis since algorithmic perfusion analysis would yield nonsensical data. This was pre-established.
Replication	Raw perfusion data was analyzed using two algorithms that are both based on vascular models but calculate output based on different imaging parameters. The first uses voxel-wise fitting of a vascular model to individual concentration-time curves obtained from the gradient echo-based DSC MRI data. The second obtained relaxation rate curves from the DSC images of the gradient echo- and another acquired dataset using spin echo-sequences. ROI placement was performed by two independent readers and intra-class correlation coefficient was

	calculated and evaluated. We also included a group of CALD patients treated with standard of care bone marrow transplant (allo-HSCT) as a positive control. However, ex-vivo our findings are based on the only available human brain sample of a patient treated with allo-HSCT. To date no additional samples (neither allo-HSCT nor gene therapy in CALD patients) are available in the NICHD Brain and Tissue Bank for Developmental Disorders.
Randomization	The STARBEAM study is a single group study, so no patients were randomized. The standard of care group was recruited from an ongoing observational cohort study. Here, all patients with the required advanced imaging protocols were included. Since we were not able to create a matching cohort, we refrained from directly comparing standard of care treatment with gene therapy. However, we performed comparisons between treated and untreated patient groups. The literature indicates that age is the most important covariate. To control for this, we drew age-matched (by nearest neighbor matching) scans from a larger imaging data base.
Blinding	For every scan ROI placement were performed blinded to perfusion and clinical data (with the exception of apparent CALD lesions on T2W imaging).

Reporting for specific materials, systems and methods

We require information from authors about some types of materials, experimental systems and methods used in many studies. Here, indicate whether each material, system or method listed is relevant to your study. If you are not sure if a list item applies to your research, read the appropriate section before selecting a response.

Materials & experimental systems

n/a	Involved in the study
☐	☒ Antibodies
☐	☒ Eukaryotic cell lines
☒	☐ Palaeontology and archaeology
☒	☐ Animals and other organisms
☐	☒ Clinical data
☒	☐ Dual use research of concern

Methods

n/a	Involved in the study
☒	☐ ChIP-seq
☒	☐ Flow cytometry
☐	☒ MRI-based neuroimaging

Antibodies

Antibodies used	- Anti-ALDP monoclonal antibody (cat# MAB2164, Chemicon)<br>- Polyclonal goat anti-human catalase antibody (cat# ab50434, Abcam)<br>- Mouse ABCD1 monoclonal antibody (cat# TA803208, Origene)<br>- Rabbit Anti-IBA1 (cat# 019-19741, Wako)<br>- Von Willebrand Factor antibody (cat# ab6994, Abcam)<br>- Goat [mouse]-1 IgG (cat# A-11001, Alexa Fluor)<br>- Goat [rabbit]-1 IgG (cat# ab150078, Alexa Fluor)
Validation	- Anti-ALDP Monoclonal Antibody : Validated by ELISA, WB, the company and reported in previous publications by immunofluorescence (Chang CL et al. 2019). We cited this antibody in Gong Y et al. 2019.<br>- Goat polyclonal Catalase antibody: Validated by ELISA, WB, IC, IH, the company and reported in previous publications by immunofluorescence (Toutzaris D et al. 2010, Gardner LB et al 2012, Hori T et al. 2013, Hori T et al. 2014).<br>- Mouse ABCD1 (Origene, TA803208): Validated by WB, IHC, the company and cited in previous publications by immunofluorescence (Sik JE 2020, Zhang Y et al. 2019). We cited this antibody in Musolino PL 2015, Gong Y et al. 2015 and Gong Y et al. 2019.<br>- Rabbit (IBA1, Wako, 019-19741): Validated by ICC, IHC(Frozen), the company and cited in 3261 previous publications.<br>- Von Willebrand Factor (Abcam, ab6994): Validated by ICC/IF, IHC-FrFl, IHC-P, IHC-Fr, WB, Flow Cyt, IHC-FoFr, the company and referenced in 385 publications.<br>- Goat [mouse]-1 IgG (H+L, Alexa Fluor® 488 conjugate, A-11001): Validated by Flow, ICC/IF, IHC ,the company and referenced in 629 publications.<br>- Goat [rabbit]-1 IgG (H+L Alexa Fluor® 555 conjugate, ab150078): Validated by IHC-Fr, ICC/IF, ELISA, IHC-P, Flow Cyt, the company and referenced in 516 publications.

Eukaryotic cell lines

Policy information about cell lines and Sex and Gender in Research

Cell line source(s)	CD34+-derived peripheral blood cells from a male patient with confirmed loss of ALDP after treatment with eli-cel.
Authentication	Loss of ALDP expression was confirmed by flow cytometry and western blot.
Mycoplasma contamination	Cell lines tested negative for mycoplasma contamination.
Commonly misidentified lines (See ICLAC register)	N/A.

Clinical data

Policy information about clinical studies
All manuscripts should comply with the ICMJEguidelines for publication of clinical research and a completedCONSORT checklist must be included with all submissions.

Clinical trial registration	ALD-102, ClinicalTrials.gov Identifier: NCT01896102
Study protocol	https://clinicaltrials.gov/ct2/show/NCT01896102
Data collection	Multicenter (United States: California, Mattel Children's Hospital UCLA/Ronald Reagan UCLA Medical Center, Los Angeles, California, United States, 90095; United States: Massachusetts, Boston Children's Hospital/Massachusetts General Hospital Boston, Massachusetts, United States, 02115; United States, Minnesota, University of Minnesota, Minneapolis, Minnesota, United States, 55455; Argentina: Medeos SRL Buenos Aires, Argentina, C1022; Australia, South Australia: Women and Children's Hospital North Adelaide, South Australia, Australia, 5006; France:Hôpital Bicêtre Le Kremlin-Bicêtre Cedex, France, 94275; Germany, University of Leipzig, Leipzig, Germany, 04103; United Kingdom Great Ormond Street Hospital for Children NHS Foundation Trust London, United Kingdom, WC1N3JH), Study start: August 21, 2013, Study end: March 26, 2021.
Outcomes	1°:<br>- Percentage of Participants Who Were Alive and Have None of the 6 Major Functional Disabilities (MFDs) at Month 24 and Without Allo-HSCT or Rescue Cell Administration [Time Frame: At Month 24] and Proportion of Participants Who Had Experienced Either Acute ([>or=] Grade II) or Chronic Graft Versus Host Disease (GVHD) by Month 24 [Time Frame: By Month 24].Proportion of Participants Who Had Experienced Either Acute ([>or=] Grade II) or Chronic Graft Versus Host Disease (GVHD) by Month 24 [Time Frame: By Month 24]<br>- Acute GVHD graded on the Acute GVHD Grading Scale (I-IV): Grade I is characterized as mild disease, Grade II as moderate, Grade III as severe (involvement of any organ system), and Grade IV as life-threatening; chronic GVHD was determined by the Investigator. Percentage of participants who experienced with either acute (>= Grade II) or chronic GVHD at Month 24 were reported.<br>2°:<br>- Percentage of Participants Who Demonstrated Resolution of Gadolinium Positivity on Magnetic Resonance Imaging (MRI) at Month 24 [Time Frame: At Month 24] Percentage of participants who demonstrated resolution of gadolinium positivity (i.e., GdE-) on MRI at Month 24 were reported.<br>- Time to Sustained Resolution of Gadolinium Positivity on MRI [Time Frame: Up to Month 24]Sustained resolution of gadolinium positivity was defined as having at least two consecutive GdE- results by MRI without a subsequent evaluation indicating GdE+.<br>- Number of Participants With Change in Total Neurologic Function Score (NFS) From Baseline up to Month 24 [Time Frame: Baseline up to Month 24].<br>- Major Functional Disability (MFD)-Free Survival Rate [Time Frame: At 24 months after Lenti-D drug infusion].<br>- Overall Survival Rate [Time Frame: At 24 months after Lenti-D drug infusion]<br>- Proportion of Participants With Neutrophil Engraftment by 42 Days Post-drug Product Infusion [Time Frame: By 42 days post-drug infusion]<br>- Time to Neutrophil Engraftment Post-drug Product Infusion [Time Frame: By 42 days post-drug infusion]<br>- Proportion of Participants With Platelet Engraftment by Month 24 [Time Frame: By Month 24]<br>- Time to Platelet Engraftment Post-drug Product Infusion [Time Frame: By Month 24]<br>- Proportion of Participants With Engraftment Failure By Month 24 [Time Frame: By Month 24]<br>- Proportion of Participants Who Underwent a Subsequent Allo-Hematopoietic Stem Cell (HSC) Infusion by Month 24 [Time Frame: By Month 24]<br>- Percentage of Participants With Transplant-related Mortality Through 100 and 365 Days Post-drug Product Infusion [Time Frame: From time of drug product infusion through 100 and 365 days post-drug product infusion].<br>- Percentage of Participants With Adverse Events (AEs), Serious AEs, Grade >=3 AE, Related AEs, Related SAEs and Related Grade >=3 AEs [Time Frame: From date of informed consent up to Month 24].<br>- Percentage of Participants With Potentially Clinical Significant Changes in Laboratory Parameters by Month 24 [Time Frame: From time of drug product infusion up to Month 24]<br>- Number of Emergency Room Visits (Post-Neutrophil Engraftment) By Month 24 [Time Frame: From Post-Neutrophil Engraftment up to Month 24]<br>- Number of In-patient Hospitalizations (Post-Neutrophil Engraftment) By Month 24 [Time Frame: From post-neutrophil engraftment up to Month 24]<br>- Duration of In-patient Hospitalizations (Post-Neutrophil Engraftment) up to Month 24 [Time Frame: From post-neutrophil engraftment up to Month 24]<br>- Number of Intensive Care Units (ICU) Stays (Post-neutrophil Engraftment) By Month 24 [Time Frame: From post-neutrophil engraftment up to Month 24]<br>- Duration of ICU Stays (Post-neutrophil Engraftment) By Month 24 [Time Frame: From post-neutrophil engraftment up to Month 24]<br>- Number of Participants With Vector-Derived Replication Competent Lentivirus (RCL) Detected by Month 24 [Time Frame: By Month 24]<br>- Number of Participants With Insertional Oncogenesis By Month 24 [Time Frame: By Month 24]

Magnetic resonance imaging

Experimental design

Design type	Not applicable.

Design specifications	Not applicable.
Behavioral performance measures	Not applicable.

Acquisition

Imaging type(s)	Structural and perfusion.
Field strength	1.5 and 3.0 T.
Sequence & imaging parameters	Perfusion imaging: Gradient echo (GRE) echo planar imaging (EPI) sequences. Acquisition parameters were TR / ST / matrix / FOV: 1500ms / 40ms / 5mm / 128x128 / 22x22cm for 1.5T and both 3T scanners. The TE was 40ms, 32ms and 35ms at 1.5T and at 3T scanners. Additional spin-echo (SE) DSC perfusion image series were acquired simultaneous to the GRE EPI sequences with a TE of 96ms and otherwise identical parameters
Area of acquisition	Whole brain scans were acquired.

Diffusion MRI ☒ Used ☐ Not used

Parameters: Axial diffusion tensor imaging (DTI; TR / TE / ST / matrix / FOV / number of directions in 1.5T: 10000ms / 87-99ms / 2.2-2.4mm / 256x256 / 22x22-cm / 25; and in 3T: 3800-9700-ms / 90-92ms / 2.0-5.0mm / 160x160 / 23x23cm / 25).

Preprocessing

Preprocessing software	Syngo.via (https://www.siemens-healthineers.com)
Normalization	Not applicable.
Normalization template	Not applicable.
Noise and artifact removal	Not applicable.
Volume censoring	Not applicable.

Statistical modeling & inference

Model type and settings	Not applicable.
Effect(s) tested	Not applicable.

Specify type of analysis: ☐ Whole brain ☒ ROI-based ☐ Both

Anatomical location(s): ROIs were placed based on previously established criteria of CALD lesion characteristics on T2-w eighted and T1-weighted post contrast maps (Musolino et al. 2012). Anatomic location varied based the primary side and location of the lesions.

Statistic type for inference (See Eklund et al. 2016)	Not applicable.
Correction	Not applicable.

Models & analysis

n/a	Involved in the study
☒	☐ Functional and/or effective connectivity
☒	☐ Graph analysis
☒	☐ Multivariate modeling or predictive analysis
